# Microplastics as vectors for microbial transport: experimental interaction with *Escherichia coli*

**DOI:** 10.1007/s00128-026-04276-1

**Published:** 2026-06-17

**Authors:** Luis Parmenio Suescún-Bolívar, Juan José Granada-Calderón, Wilkendry Ramos Cervantes, John Betancourt, Jesús E. Diosa, Edgar Mosquera-Vargas

**Affiliations:** 1 Licenciatura en Educación con Énfasis en Ciencias Sociales y Ambientales, Universidad de Cartagena, Cartagena de Indias, 130001 Colombia; 2 Biodiversity, Molecular and Genetic (BioMolGen) foundation, Ciudad de México, México; 3 Fundación para la Investigación en Inmunología, Farmacología y Toxicología (FIIFT), Cartagena de Indias, Colombia; 4 Facultad de Ciencias Básicas, Departamento de Microbiología, Universidad Santiago de Cali, Santiago de Cali, Colombia; 5 Institución Educativa Número Dos, Maicao, 442001 Colombia; 6 Grupo de Investigación Catálisis y Materiales, Programa de Química, Facultad de Ciencias Básicas y Aplicadas, Universidad de La Guajira, Riohacha, 440007 Colombia; 7 Grupo de Transiciones de Fase y Materiales Funcionales, Departamento de Física, Universidad del Valle, Santiago de Cali, 760032 Colombia; 8 Centro de Excelencia en Nuevos Materiales (CENM), Universidad del Valle, Santiago de Cali, 760032 Colombia

**Keywords:** Microplastics (MPs), *E. coli*, Vibrational spectroscopy, Microscopy characterization

## Abstract

Microplastics (MPs) are recognized as vectors for microorganisms in aquatic ecosystems, raising concerns about their environmental implications. We examine the structural and chemical properties of recycled PET microplastics and their interactions with *Escherichia coli* (*E. coli*) using Fourier-transform infrared (FTIR) and Raman spectroscopy. FTIR analysis identified nine characteristic vibrational bands of PET, and Raman spectroscopy confirmed that neither glutaraldehyde treatment nor bacterial exposure produced significant chemical changes in the PET structure. Scanning electron microscopy (SEM) and Gram staining revealed bacterial adhesion and biofilm formation on microplastic surfaces. Additionally, *E. coli* colonies exhibited reduced lactose fermentation activity. These findings reinforce the role of MPs as microbial vectors and demonstrate the ability of *E. coli* to colonize synthetic polymers. The development of rapid spectroscopic tools could enhance monitoring efforts in both laboratory and field environments. This study contributes to the growing understanding of MPs-microorganism interactions, and a model system of MPs-microorganism interaction is proposed.

## Introduction

Aquatic ecosystems act as major sinks and transport pathways for plastic particles less than 5 mm, known as microplastics (MPs), which enter through diverse anthropogenic pathways and persist due to their resistance to degradation. Their widespread distribution across atmosphere, water columns, sediments, and organisms makes MPs a critical component of environmental contamination (Amelia et al. 2021). They originate either from direct industrial production (primary MPs) or from the degradation of larger plastic debris (secondary MPs) through physical and chemical processes (Duis and Coors 2016). Beyond their physical accumulation, MPs modify surrounding physicochemical conditions (as pH, soil respiration, metabolic efficiency) and disrupt microbial communities and key ecosystem processes like surface-driven microbial selection, alteration of nutrient cycling, biofilm-mediated ecological shifts (Zhao et al. 2021; Suescún-Bolívar et al. 2025). Of toxicological relevance is their ability to adsorb pharmaceuticals, antibiotics, and other hazardous compounds, thereby enhancing pollutant mobility and bioavailability (Wu et al. 2022). This carrier function increases ecological exposure to complex contaminant mixtures and contributes to emerging risks such as antimicrobial resistance, positioning MPs as active agents in aquatic toxicology rather than inert debris.

Microplastics also provide stable substrates for microbial colonization, including potentially pathogenic *Enterobacterales* such as *Escherichia coli* (*E. Coli*) (Oberbeckmann et al. 2018; Gong et al. 2019; Octavia and Lan 2014). Experimental evidence indicates that MP exposure can alter bacterial metabolism, promote biofilm formation, and facilitate the persistence and dissemination of pathogens and resistance genes (Yi et al. 2021; Gao et al. 2021; Feng et al. 2023). Although some key mechanisms of MP–microorganism interactions have been described (Yan et al. 2024), effective experimental model systems remain limited, particularly for studying early microbial adhesion, gene transfer, MPs degradation, and contaminant adsorption. Addressing this gap, the present study investigates the ability of *E. coli* to colonize MPs generated from PET bottles under controlled conditions, and the possible structural and chemical changes in PET microplastics interacting with *E. coli* using vibrational spectroscopy techniques and scanning electron microscopy (SEM). We used PET MPs because PET is one of the most widely produced polymers globally, due to its use in beverage bottles and textiles, leading to its frequent detection in aquatic environments, particularly as secondary MPs derived from consumer waste (Geyer et al., 2017). Although MPs such as polyethylene (PE) and polypropylene (PP) are also abundant in the environment and are highly hydrophobic and chemically inert, PET contains ester functional groups that confer greater surface polarity and facilitate stronger interactions with water, organic matter, and microbial cells, thereby enhancing biofilm formation and microbial colonization. (Oberbeckmann et al. 2018; Amaral-Zettler et al. 2020).The proposed experimental model system offers a valuable framework for evaluating MP-associated toxicity, microbial transport, and contaminant interactions, contributing to improved risk assessment and management strategies in environmental and water treatment contexts.

## Materials and Methods

To obtain microplastic particles smaller than 5 mm recycled polyethylene terephthalate (PET) water bottles were thoroughly cleaned and disinfected using a 70% methanol solution. Following this cleaning step, the bottles were manually scraped with a cutter to separate the plastic. The resulting material was then filtered through a 35-mesh (0.5 μm) filter to isolate the desired microparticles. The recovered particles in the filters with 3.1 mm grids did not exceed the grid size and were confirmed through SEM and micro-Raman imaging. To remove any remaining organic matter, the microparticles were treated with a 6% hydrogen peroxide solution for 6 min, with agitation every 2 min. After treatment, the MPs air-dried in a laminar flow cabin under sterile conditions and stored in sealed sterile Eppendorf tubes to prevent contamination, until further analysis. All reagents and consumables were of laboratory grade and used as received.

Vibrational spectroscopy (FTIR and Raman) was used to characterize MPs (Fernandes et al. 2026; Xu et al. 2019) (~ 800 μm to ~ 3 mm) from recycled PET water bottles (with or without glutaraldehyde) and from *E. coli* culture media preserved in glutaraldehyde to assay any possible structural and chemical changes in PET microplastics-*E. coli* interaction. Infrared spectra were obtained using a JASCO FT/IR-6800 Type A Spectrometer equipped with an ATR Pro One module and a Tri Glycine Sulphate (TGS) detector. Each spectrum was recorded with 64 scans (with automatic baseline correction) over a range of 3500 to 500 cm^–1^ at a resolution of 4 cm^–1^.

Raman spectra, on the other hand, were recorded using a JASCO NRS-4500 Confocal Raman spectrometer equipped with a 785 nm excitation source. The excitation light was focused onto the sample through a 100× objective lens at room temperature (RT). The laser excitation power on the sample was 0.3 mW. The measurement time for each sample was 30 s, with 10 accumulations to facilitate the visualization of the characteristic vibrational modes of PET. Data was acquired in the range of 500 to 1800 cm^–1^. Raman spectral positions were calibrated using the Si peak position at 520 cm^− 1^ (not shown).

In this study, the bacteria *Escherichia coli* (Gram-negative *E. coli*, ATCC 25922) was used as a model strain. *E. coli* was cultivated for 8 h in a liquid nutritive culture media (ALPHA, Batch 190808042308) at 37 °C under constant agitation at 120 rpm before the experiment. Additionally, to carry out our test with MPs, around 20 MPs particles were added to *E. coli* culture (treatment), and the assays were incubated for 24 h at 37 °C while maintaining constant agitation. Using the same procedure, 20 MPs particles were added to a nutritive media without bacteria as a control parameter. All experiments (treatment and control assays) were conducted in triplicate.

To assess whether the *E. coli* cells adhered to the MPs, we performed the following procedures: (i) Treatment and control culture media were plated in triplicate directly on Eosin Methylene Blue (EMB) agar; (ii) additionally, five MPs were collected using 0.45 μm filters, thoroughly washed five times with sterile distilled water, and then plated in triplicate on EMB agar. All cultures were incubated for 24 h at 37 °C. After incubation, the grown colonies were characterized both macroscopically and microscopically.

Macroscopic evaluation was performed by direct observation of metallic green colonies on EMB agar in both treatment and control groups. Instead, microscopic evaluation involved Gram staining, followed by observation under an Advanced Optical microscope at 1000× magnification to identify Gram-negative bacillus (Bartholomew and Mittwer 1952).

On the other hand, we examined the presence of bacillus on MPs using a Phenom Pro X scanning electron microscope (SEM). Some MPs obtained from recycled PET water bottles and *E. coli* culture media were stored in 5% glutaraldehyde (GA, 25% solution in water, Sigma Aldrich, USA) at 4 °C until further analysis. After drying by capillary action on absorbent paper, the samples were prepared for SEM analysis. A thin layer of gold was sputter-coated onto the MPs surface to enhance conductivity. The SEM was operated at an accelerating voltage of 15 kV.

### Data Analysis and Quality Control

All experiments and control assays were performed in triplicate to ensure reproducibility of the results. FTIR and Raman spectra from PET MPs exposed and non-exposed to *E. coli* were compared to identify possible structural or chemical modifications.

Microbiological characterization included the macroscopic evaluation of colonies on EMB agar and microscopic examination following Gram staining. Bacterial colonies and MP particles were randomly selected to ensure a representative evaluation of the samples.

SEM characterization allowed the qualitative assessment of bacterial adhesion on the surface of PET MPs. All samples were prepared and analyzed under identical experimental and instrumental conditions to minimize analytical variability.

Quality assurance and quality control measures included the use of sterile materials, standardized incubation conditions, instrumental calibration prior to spectral acquisition, and bacteria-free controls to identify potential contamination.

## Results and Discussion

**Fig. 1:**
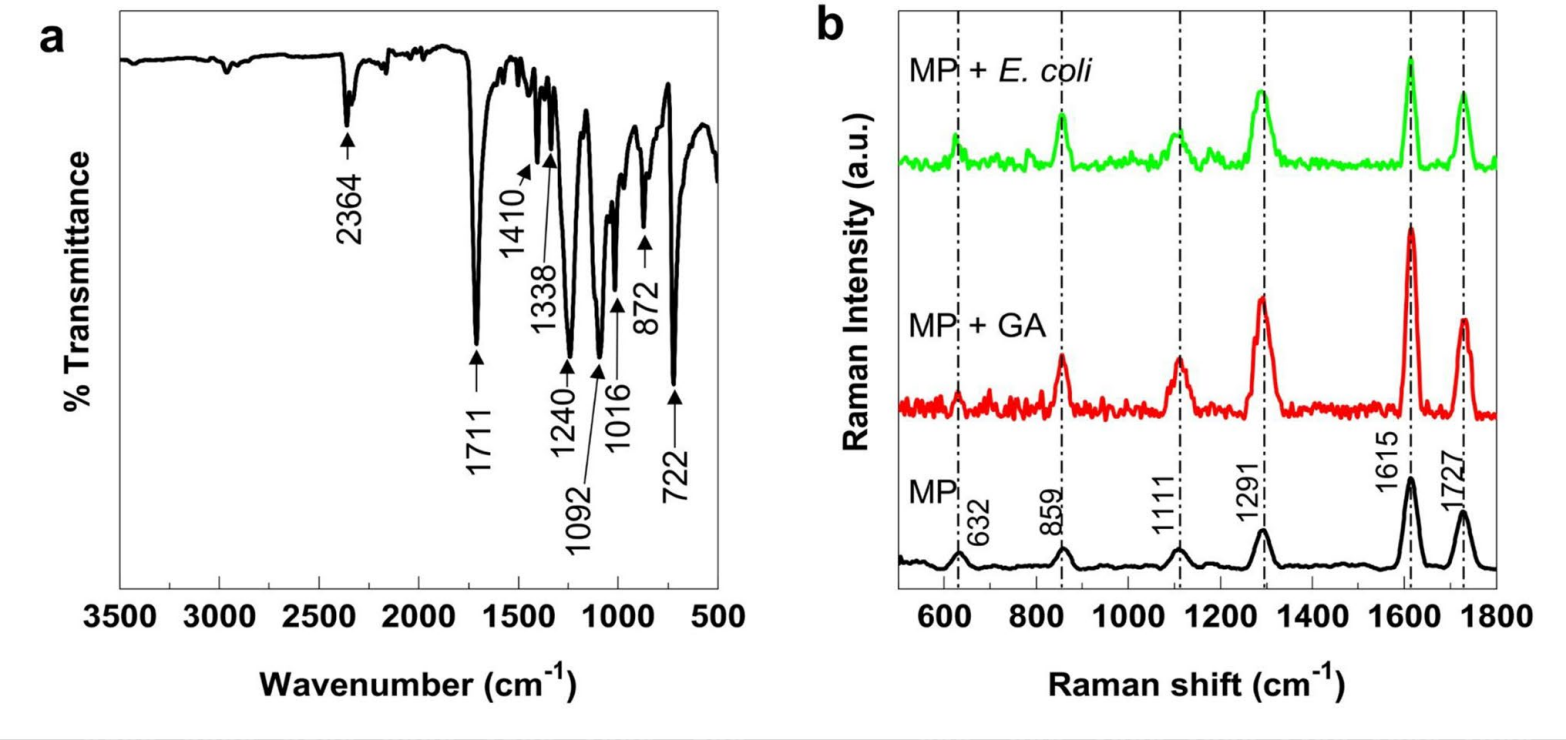
Vibrational spectroscopy. **a** FTIR spectrum of recycled PET water bottles (MPs). **b** Raman spectra of all analyzed samples: Microplastic (black), microplastic with GA (red), and microplastic with *E. coli* (green)

The FTIR absorbance spectrum serves as a structural fingerprint for the identification and chemical characterization of PET, even when variations in peak positions are present. Such variations may arise from the source of the material or its degree of aging (Guo et al. 2022). Nevertheless, this technique captures the optical responses of surface functional groups and provides critical insights into the chemical characterization of materials. Figure 1a presents the FTIR spectrum of recycled PET water bottles (MPs), highlighting their spectral features. Analysis of the obtained spectra reveals that significant signals from a specific microplastic may coincide with peaks from other MPs at certain wavenumbers. Furthermore, in samples containing multiple plastic components, some signals may overlap or appear close. As a result, using these overlapping peaks with high intensity for microplastic identification may lead to misinterpretations and reduced accuracy.

To determine the characteristic wavenumbers of the analyzed microplastic, a sample was prepared and examined using FTIR spectroscopy. A total of nine vibrational bands were identified at 722, 872, 1016, 1092, 1240, 1338, 1410, 1711, and 2364 cm⁻¹. The band at 1711 cm⁻¹ is attributed to ester carbonyl bond stretching (Dodi et al. 2022), while the band at 1410 cm⁻¹ corresponds to C–H bending vibrations (Ozaltin et al. 2019). The vibrational mode at 1338 cm⁻¹ is associated with -CH_2_ wagging (Mecozzi and Nisini 2019; Ozaltin et al. 2019).

Additionally, the bands at 1240 and 1092 cm⁻¹ correspond to C–C–O asymmetric stretching and O–C–C stretching, respectively. The band observed at 1016 cm⁻¹ is assigned to C–H in-plane vibrations, whereas the band at 872 cm⁻¹ is related to C–H out-of-plane bending (ring). The band at 722 cm⁻¹ is attributed to the interaction between ester groups and benzene rings (Andanson and Kazarian 2008; Dimassi et al. 2023; Donelli et al. 2009; Mecozzi and Nisini 2019). Lastly, the band at 2364 cm⁻¹ corresponds to atmospheric CO₂, a common background contaminant present during FTIR-ATR spectroscopic analysis (Schott et al. 2021).

The chemical structure of recycled PET water bottles (MPs without and with glutaraldehyde (GA) as well as *E. coli* culture media preserved in GA has been studied using Raman spectroscopy (Fig. 1b**)**. The Raman spectra of the recycled MPs sample exhibited the characteristic fundamental vibrational modes of PET material (black spectrum in Fig. 1b), which have been extensively reported in literature (Feng et al. 2023; González-Córdova et al. 2023; Jin et al. 2022; Peñalver et al. 2023; Zhang et al. 2023). These bands are summarized in Table 1.

Six characteristic bands are observed at 632, 859, 1111, 1291, 1615 and 1727 cm^–1^. The very strong (vs.) band located at 1615 cm^–1^ corresponds to C = C aromatic stretching, while the intense bands located at 1291 and 1727 cm^–1^ are associated with C–C stretching and C = O (ester) stretching, respectively. The band at 1111 cm^–1^ corresponds to C-O-C asymmetric and symmetric stretching. The band at 633 cm^–1^ is associated with C–C–C in-plane bending (ring). The band at 859 cm^− 1^ corresponds to C–C stretching (ring breathing) and C–O stretching. It is important to note that no additional vibrational modes were visually perceptible in the recycled PET microplastic, indicating no significant structural modifications.

**Table 1:** Raman assignment of the vibrational modes for recycled-PET microplastic

Raman shift (cm^–1^)	Assignment of the vibrational mode
1727 (s)	C = O stretching (ester)
1615 (vs.)	C = C aromatic stretching
1291 (s)	C–C stretching
1111 (m)	C–O–C asymmetric and symmetric stretching
859 (m)	C–C stretching (ring breathing) and C–O stretching
632 (m)	C–C–C in-plane bending (ring)

vs.: strong; s: strong; m: medium

On the other hand, the Raman spectrum of the GA-treated microplastic sample (red spectrum in Fig. 1b) showed changes in the relative intensity of the peaks, compared to the untreated microplastic (black spectrum). However, no shifts were observed in the positions of the characteristic peaks, suggesting that GA did not induce any noticeable chemical alterations in the PET microplastic structure.

Previous studies by Bik et al. (2020) demonstrated that GA fixation in solution can influence the Raman spectrum by increasing or decreasing the Raman signal due to interactions with cellular components. In our study, the observed differences may be more related to physical surface changes or optical effects rather than direct chemical modifications of the polymer.

Similarly, no vibrational changes were observed in the microplastic sample containing *E. coli* bacteria (green spectrum in Fig. 1b). This indicates that the presence of bacterial cultures did not significantly affect the Raman signal of the microplastic, reinforcing the conclusion that neither GA treatment nor bacterial exposure induced detectable chemical modifications in the PET structure under the experimental conditions used.

**Fig. 2:**
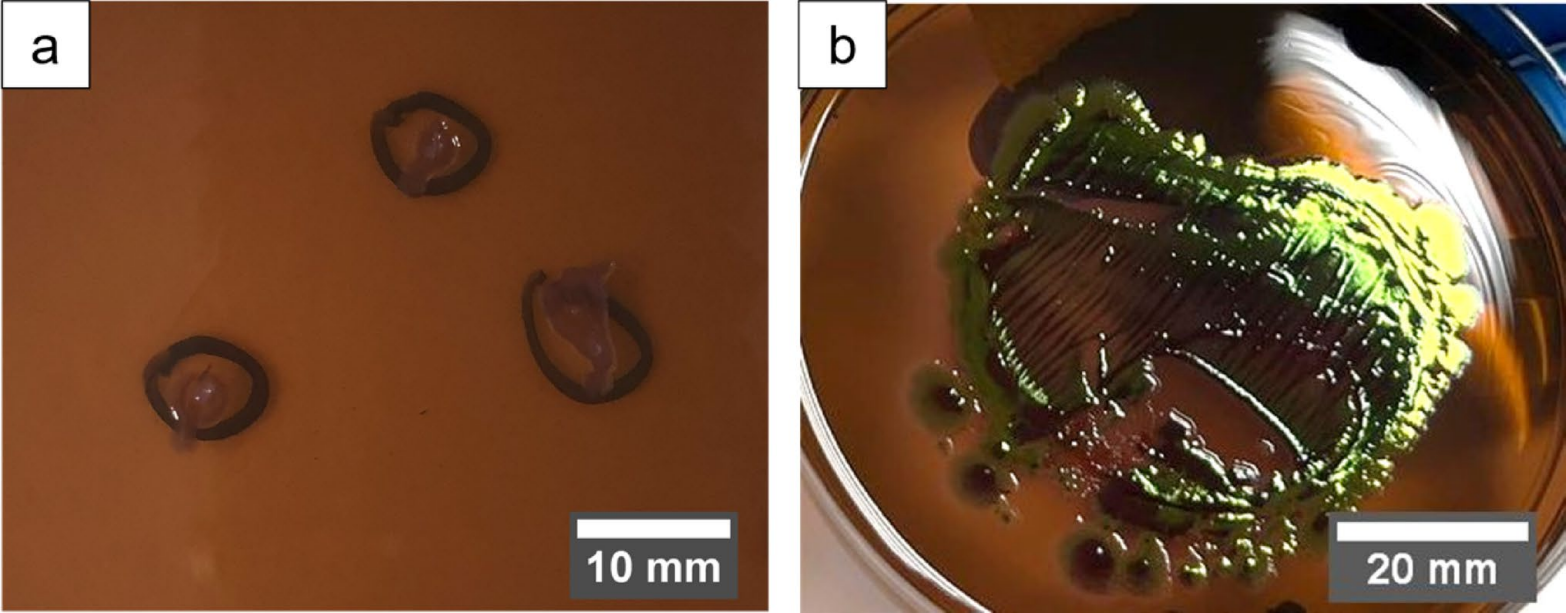
Photographs of three microplastic particles **a** and liquid culture bacteria **b** plated on a Petri dish containing EMB medium. In **b**, note the characteristic metallic green color indicative of lactose fermentation

The MPs recovered from liquid cultures of *E. coli* and subsequently inoculated onto solid EMB media produced creamy white to transparent colonies (Fig. 2a). A clear proliferation of adhered bacterial colonies on the microplastic surface was observed, as evidenced by the progressive expansion of an unpigmented area on the Petri dish. However, these colonies did not exhibit the characteristic metallic green color associated with lactose fermentation (Fig. 2b).

**Fig. 3:**
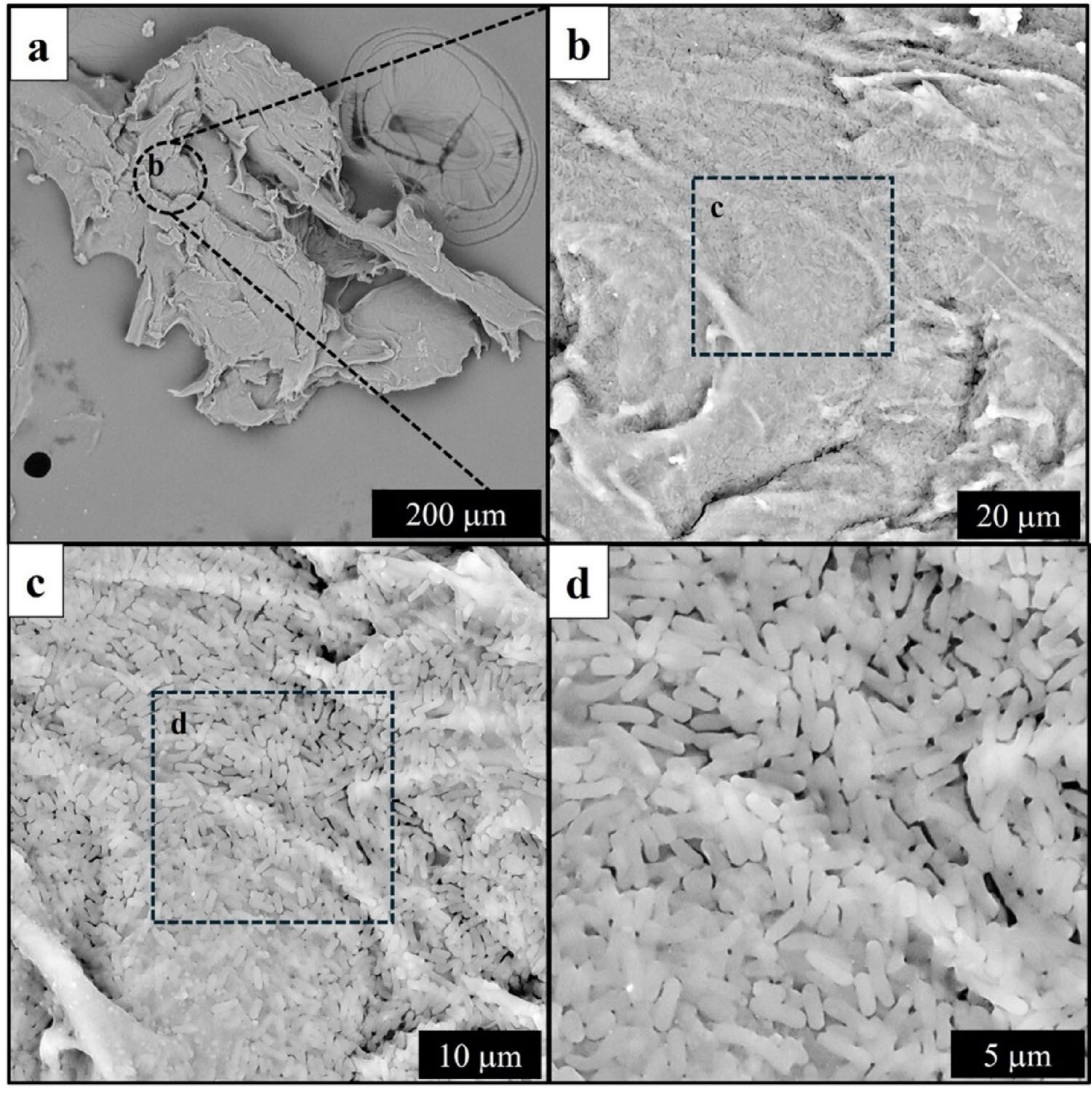
SEM images of a microplastic (MP, ca. 800 μm) at different magnifications after plating on EMB agar medium. **a** Displays the MP with the inset showing *E. coli* bacteria. **b**, **c** and **d** show high magnification scanning electron micrographs revealing the presence of *E. coli* bacteria on MP surfaces. Insets in **b** and **c** provide even higher magnification corresponding to the areas previously shown in the main micrographs

Scanning electron microscopy (SEM) imaging revealed the morphology of microplastic particles following plating on EMB agar medium (Figs. 3a-d). A microplastic particle of approximately 800 μm in size was observed (Fig. 3a), displaying an irregular surface characterized by cracks and cavities throughout its structure. Notably, rod-shaped bacteria were visibly attached to and colonizing the microplastic surface (Fig. 3b-d). These bacteria, with an average diameter of 0.60 (± 0.05) µm and a length of 1.7 (± 0.3) µm, densely covered the microplastic, forming a biofilm. These findings highlight the adaptability of *E. coli* and provide valuable insights into the complex interactions between microbes and MPs.

The results confirm that *E. coli* bacilli can successfully colonize PET microplastics under experimental conditions. Furthermore, the observed loss of lactose fermentation capability suggests a possible metabolic shift, wherein *E. coli* may utilize microplastic-associated compounds as an alternative carbon source. Previous studies have demonstrated that bacterial retention on MPs is influenced by particle size and concentration (Gao et al. 2021) as well as the absorption of nutrients by MPs (Han et al. 2013) or the release of plastic-derived compounds (Chen et al. 2021). Thus, it is highly plausible that the bacteria in this study exploited nutrients retained by MPs during exposure to liquid cultures, supporting the role of *E. coli* as a potential pioneer microorganism in microplastic colonization (Gong et al. 2019; Oberbeckmann et al. 2018).

Our laboratory-based findings confirm that *E. coli* can successfully adhere to and colonize PET MPs forming dense biofilms, but it was not possible to determine the MP-bacteria interaction through spectroscopic changes by FTIR or Raman analysis. The absence of lactose fermentation activity in *E. coli* colonies suggests a possible metabolic adaptation, potentially utilizing MP-associated compounds as an alternative carbon source. This highlights the complex interactions between microplastics and microbial communities, reinforcing the role of MPs as carriers of microorganisms in aquatic, soil and atmospheric environments. This fact could facilitate the transport and dispersal of this enterobacterium, posing a threat to public and environmental health (Sun et al., 2021; Octavia and Lan 2014). Furthermore, *E. coli*, as a member of the order Enterobacterales (Gong et al. 2019; Oberbeckmann et al. 2018), under environmental conditions could be affect the structure of the microbial community of the “plastisphere,” which includes a wide variety of bacteria, archaea, microalgae, and fungi (Agostini et al. 2021; Zettler et al. 2013). Thus, it is necessary to conduct interaction studies about microplastics with multi-species microorganisms in environmentally realistic conditions.

On the other hand, MPs may act as reservoirs for antibiotic resistance genes, promoting their selection and horizontal transfer, which exacerbates the growing issue of antimicrobial resistance. High concentrations of MPs in aquatic environments also facilitate the retention and deposition of *E. coli*, prolonging its persistence and increasing its potential for wider spread across ecosystems (Gao et al. 2021). This extended survival on MP surfaces allows pathogenic strains to remain viable for longer, raising concerns about the role of MPs in transporting harmful bacteria and their implications for waterborne diseases.

Further research is required to explore the broader implications of MP-microorganism interactions under environmentally relevant conditions. Future studies should investigate the influence of MP size, shape, and chemical composition on microbial colonization, as well as the potential ecological succession of microbial communities on MPs. Additionally, incorporating nutrient availability as a variable will enhance our understanding of microbial adaptation and persistence on MPs.

A limitation of this study was the absence of a spectrophotometric signature to confirm bacterial presence on MPs due to insufficient blank measurements. Addressing this issue in future research will be essential for developing reliable spectroscopic tools for rapid detection of microbial colonization. Advancing these diagnostic techniques will facilitate the monitoring of MP-associated microbial communities, improving risk assessment and management strategies in both laboratory and field settings.
